# Recognize the role of CD146/MCAM in the osteosarcoma progression: an in vitro study

**DOI:** 10.1186/s12935-021-02006-7

**Published:** 2021-06-08

**Authors:** Xing Lei, Kewei Wang, Wenbo Wang, Hao Jin, Wenguang Gu, Zhiguo Chen, Wei Wang, Kaituo Gao, Huan Wang

**Affiliations:** 1 Department of Orthopedic Surgery, Linyi People’s Hospital, Linyi, 276000 China; 2 Center for Endemic Disease Control, Chinese Center for Disease Control and Prevention, Harbin Medical University, 157 Baojian Road, Harbin, 150081 China; 3 Department of Orthopedic Surgery, The First Affiliated Hospital, Harbin Medical University, 23 Youzheng Street, Nangang District, Harbin, 150001 China

**Keywords:** Osteosarcoma, CD146/MCAM, Endothelium, Migration, High glucose

## Abstract

**Background:**

Osteosarcoma (OS) is a common malignant bone tumor with poor prognosis. We previously reviewed that CD146 is correlated with multiple cancer progression, while its impact on OS is currently not systematically studied.

**Methods:**

MG63 was transfected with lentivirus to express CD146 ectopically, and anti-CD146 neutralizing antibody ab75769 was used to inhibit 143B. Cyclic migration of MG63 and co-culture between MG63 and 143B were used to explore the role of OS malignancy in CD146 expression. The effect of OS cell medium (CM) on endothelium behaviors was assessed, and the expression changes of CD146 before and after co-culture of endothelium and OS were evaluated. Finally, the expression of CD146 in OS was detected under different culture conditions, including hyperoxia, low oxygen, high glucose and low glucose conditions.

**Results:**

CD146 promoted the colony formation, migration, invasion and homotypic adhesion of OS cells, and reducing the concentration of soluble CD146 in the OS medium inhibited the proliferation, migration and lumen formation of the cultured endothelium. However, CD146 did not affect the adhesion between OS and endothelium, nor did co-culture of both sides affect the CD146 expression. Similarly, the proliferation, migration and CD146 expression of MG63 remained unchanged after many cycles of migration itself, as did its co-culture with 143B for expressing CD146. In addition, we also showed that high glucose promoted the expression of CD146 in OS, while hypoxia had the opposite effect.

**Conclusions:**

These findings demonstrate that CD146 promotes OS progression by mediating pro-tumoral and angiogenic effects. Thus, CD146 could be a potential therapeutic target for OS, especially for OS patients with diabetes.

**Supplementary Information:**

The online version contains supplementary material available at 10.1186/s12935-021-02006-7.

## Background

Osteosarcoma (OS) is the most common primary non-hematopoietic malignant bone tumor in children and adolescents (estimated incidence: 4.8/million/year) [1, 2]. Although various efforts have been exerted during the past decades, the prognosis of patients with OS remains poor due to its characteristics of migration, invasion and early lung metastasis [3]. Therefore, targeted inhibition of certain molecules closely related to OS progression may be one of the effective ways to solve the above problem.CD146, also known as MCAM, is a member of the immunoglobulin superfamily [4, 5]. Our previous study reviewed that CD146 was closely associated with the progression of several cancers, including melanoma, prostate cancer, breast cancer, etc. [6]. In 2003, McGary and co-workers first showed that CD146 was widely expressed on OS cells, such as TE-85, SaOS-2, MNNG/HOS and KRIB cells, but MG63. The authors also found ABX-MA1, a specific CD146 mAb, could inhibit the adhesion, invasion and lung metastasis of the KRIB cells [7], but there is no research involving cell cloning, migration and proliferation. In addition, the authors and other later researchers have not explored the effect of CD146 on the other OS cells. Thus, the effect of CD146 on OS progression in vitro is not very clear, and vice versa.

Angiogenesis is essential for tumor growth. CD146 is widely expressed in vascular system [8], and plays an important role in angiogenesis, vascular permeability and leukocyte migration [9, 10], involving VEGFR2 pathway [11, 12] and its interaction with netrin-1 [5, 13]. Soluble CD146 (sCD146) is a new factor with angiogenic properties [14], which is generated through the shedding of the extracellular portion of CD146 [15]. The sCD146 participates in the angiogenesis process through the interaction with angiogenin, which is an important regulator of endothelial cell migration and tube formation [16]. Therefore, whether OS cells affect endothelial function by secreting sCD146 to regulate their own nutrient supply is also a question of our concern. Due to the imbalance of vascular flow and distribution in tumor tissue, tumor is often in a state of hypoxia, extracting only 5–50% of the total oxygen content in arterial blood [17, 18]. In addition, increasing evidence suggests a complex relationship between diabetes and cancer. Patients with type 2 diabetes have an increased risk of multiple cancers, especially pancreatic cancer and liver cancer [19], and a coincidence of cancer and diabetes worsens outcome and increases mortality [20]. Thus, after knowing whether the expression of CD146 interacts with the progression of OS, it is necessary to know the changes of CD146 expression of tumor cells under hyperoxia (HO), low oxygen (LO), high glucose (HG) and low glucose (LG) conditions, so as to provide a reference for predicting the potential internal relationship and prognosis between tumor and various complications.

In this study, we selected two kinds of OS cells with great different malignancy, MG63 (low) and 143B (high) [21], and human umbilical vein endothelial cells (HUVECs) to explore the role of CD146 in the OS progression and the interaction between OS and endothelial cells in vitro. The results showed that CD146 promoted the progression of OS by increasing the invasion, migration and proliferation activity of OS cells. Unexpectedly, the proliferation and migration of MG63 OS cells were not enhanced after many cycles of migration in vitro, and the expression of CD146 did not turn positive, either. Compared with the natural endothelial cell medium (CM), HUVECs cultured in OS CM could not increase its migration, invasion and tube-like structure, but the decreased concentration of sCD146 in the CM significantly inhibited the above mentioned effects. Moreover, the expression of CD146 in OS responded differently to different culture environments. Our data suggest that CD146 has an important role in the prognosis of OS and may be a novel therapeutic target for OS treatment.

## Material and methods

### Lentivirus transfection

MG63 cells (1 × 10^6^) were seeded to each 60-mm petri dish (about 80% confluence) 1 day before transfection. A standard transfection procedure from the manufacturer (Life Technology) was followed. The cDNA coding region to human CD146 was PCR amplified and subcloned into the lentiviral shuttle vector pwpxl. Lentiviruses were produced in HEK293 cells and amplified to obtain high titers, G418 (0.25 mg/ml; active component 0.19 mg/ml, LD50) was added for screening G418R clones. Each clone was expanded and huMUC18 expression was determined by Western blot analysis, resulting in the lentivirus expressing CD146 (Lenti-CD146) that also expressed GFP as a marker to monitor infection efficiency. Lenti-GFP was used as a control.

### Cell culture

All cell lines were purchased from the American Type Culture Collection (Manassas, VA). A375SM, SB-2 melanoma and U2OS OS cell lines were cultured in Dulbecco’s modified Eagle’s medium (DMEM) (Gibco Life Technologies) with 1 g/l glucose and 10% fetal calf serum (FCS). MG63, 143B, Lenti-CD146, Lenti-GFP and Human umbilical vein endothelial cells (HUVECs) were propagated in RPMI 1640 medium (Gibco Life Technologies) supplemented with 10% FCS. All of the cells were incubated at 37 °C in conditions of 5% CO_2_.

### Immunofluorescence

All kinds of OS cells were plated on confocal dishes in advance 24 h, respectively. The cells were washed 3 times with phosphate-buffered saline, fixed in 4% paraformaldehyde, and permeabilized with 0.2% Triton X-100. After being washed with PBS and incubated with blocking reagent (5% nonfat milk in PBS) for 15 min, cells were incubated for 1 h at room temperature with anti-CD146 mAb ab75769 (Abcam, Cambridge, MA, USA) or irrelevant IgG1 mAb (1:500). Secondary labeling was then done for 2 h with PE sheep anti-rabbit IgG (1:100). The nucleus was stained by DAPI (10 μg/ml, Invitrogen) for 15 min at room temperature. Samples were examined with a confocal laser scanning microscope (Olympus, Tokyo, Japan), and using Image J software (National Institutes of Health, USA) merged images.

### Western blotting

All kinds of cell lines were washed with PBS and harvested in RIPA buffer. Total protein concentrations were measured using the bicinchoninic acid assay (Pierce Chemical Co Rockford, IL, USA). Samples were loaded at 40 μg/lane and separated on 8–12% SDS–polyacrylamide gels and then transferred to polyvinylidene difluoride membranes and probed with specific primary anti-CD146 mAb ab75769 or ab134065 (Abcam, Cambridge, MA, USA). To detect the signal, peroxidase-conjugated secondary antibody was added, followed by exposure using enhanced chemiluminescence (Amersham, Arlington Heights, IL, USA). The intensity of the amplified products was quantified by densitometry analysis and referred to that obtained with glyceraldehyde-3-phosphate dehydrogenase (GAPDH). Densitometry analysis was performed by Image J software (National Institutes of Health, USA) on at least three independent experiments.

### RNA extraction, semi-quantitative (sq) and real-time qPCR assay

Total RNAs were extracted from cells using Trizol solution (Invitrogen), according to the manufacturer’s instructions. RNA concentrations were measured using the Nanodrop2000 spectrophotometer. Complementary DNA (cDNA) was synthesized from RNA using a PrimeScript RT reagent kit (TaKaRa, Japan). Semi-quantitative PCR (sq-PCR)was carried out to determine the gene expression levels of CD146 and GAPDH in human MG63, A375SM, and HUVEC cells, while real-time quantitative PCR (qPCR) was used to assess expression of CD146 and GAPDH in all kinds of cells researched. The generation of specific PCR products was confirmed with melting-curve analysis, and data presented as target gene expression normalized to GAPDH. According to the sequence of CD146 mRNA (NCBI Reference Sequence: NM-006500.2), CD146 primers were designed by using Premier Primer 5 software (Premier, Canada). The primers used were shown in Table 1.

**Table 1:** Sequences of primers for the PCR analysis

Gene	Primer 5′–3′	Fragment (bp)
CD146	F: AGTCCTGAGCACCCTGAATGTCC	263
	R: CAATCACAGCCACGATGACCAC	
GAPDH	F: CCTCTGACTTCAACAGCGACAC	174
	R: TGGTCCAGGGGTCTTACTCC	

### Cell migration and invasion assay

Before migration, cells were serum-starved overnight. Then, 2 × 10^5^ OS cells or 1 × 10^6^ HUVECs in 200 μl of the serum-free RPMI 1640 medium were seeded to each top well of a 12-well Transwell Boyden system (8 μm pore size, Sigma, CLS3422), and 500 μl RPMI 1640 medium supplemented with 10% fetal calf serum was added to the lower chamber. Cells were allowed to migrate for 24 h at 37 °C, in 5% CO_2_. After removing cells on the upper surface of the filter using cotton swabs, cells that invaded through the membrane were fixed with 4% paraformaldehyde for 20 min and stained with 0.1% crystal violet solution for 15–20 min. The number of cells that reached the lower part of the Transwell filter membrane was counted with Image J software (National Institutes of Health, USA) and plotted as the number of cells per optic field (× 200). Experiments were carried out in triplicate.

The invasive procedure is almost the same as the migration assay except for the 8 μm pore size (corning, CLS3422) coated with 150 μg of Matrigel (65 μl of 2.3 mg/ml of Matrigel, Becton Dickinson Matrigel Basement membrane Matrix, phenol-red free, Collaborative Research Cat. no. 40234C) used.

### Cyclic migration assay (Additional file 1: Fig. S1)

5 × 10^5^ MG63 cells in 0.1 ml of the serum-free RPMI 1640 medium were seeded to each top of Transwell chamber. After migration for 48 h at 37 °C, in 5% CO_2_, cells that migrated through the membrane were collected and then amplified culture. When the number of cells is sufficient, the migration operation is carried out again according to the steps mentioned above until the 9th or 15th time. The cells prior to migration (i.e., 0th), migration 9th and 15th were cryopreserved in a − 80 °C refrigerator for subsequent cell migration, proliferation, PCR and Western Blotting detection. Experiments were carried out in triplicate.

### Matrix colony formation assay

The BD matrix (60 μg/ml; BD, USA) and the medium were diluted with 1:3 and added to the 6-well plate. The bottom layer of the gel was poured and allowed to solidify after incubation with 4 h at 37 °C, in 5% CO_2_. The cells were laid in each well at 1 × 10^4^/ml concentrations and cultured for more than 14 days. The culture process was not terminated until small clones were visible to the naked eye in the plate. After discarding the supernatant, the clones were washed twice with PBS carefully and fixed with 4% paraformaldehyde for 15 min and stained with 0.4% crystal violet solution for 10 min. The number of clones counted in a microscope (× 100) was greater than 20 cells. The clone formation rate was calculated: clone formation rate = (number of clones/number of inoculated cells) × 100%.

### Cell proliferation assay

143B cells (2.5 × 10^4^/ml, 200 μl//well in 96-well plates) were treated with 1, 2, 4, 8 μg/ml ab75769 or IgG control Ab for 0 h, 6 h, 12 h, 24 h, 36 h, 48 h respectively, then analyzed for viability by MTT assay to explore the optimal inhibitory concentration of antibodies against cells. The most efficient inhibitory concentration group and the control group were defined as “ab75769 group” and “IgG group”, respectively, and follow-up experiments were conducted.

143B cells (ab75769 and IgG group), HUVECs, Lenti-CD146 MG63 or pwpxl vector-only transfected MG63 cells were seeded on 96-well plates at a density of 1 × 10^4^/ml, 200 μl/well, and cultured in medium for indicated time points. The colorimetric MTT assay was performed as indicated by the manufacturer. Absorbance was read at 490 nm. Experiments were performed at six times.

Another method of detecting cell proliferation, which we called Cell counting assay, was determined by plating cells (1 × 10^4^/ml, 500 μl//well) into 24-well plates and cultured in medium. The subconfluent cells were then collected by trypsinization at the indicated time point. The number of viable cells, as determined by trypan blue staining, was counted at the indicated time points at least three times.

It should be noted that the culture medium (CM) used to detect endothelial proliferation was derived from the CM for OS cells, as shown in the “Endothelial cells were cultured in OS CM” section.

### Adhesion of OS cells to extracellular matrix (ECM)

96-well dishes were coated with 0.2% type I collagen (Stemcell, Canada, 100 μl/well). The collagen was air dried to the surface, and the plate was incubated with 1% BSA for 2 h to block irrelevant attachment sites. OS cells (5 × 10^5^/ml) were added to each well with and without ab75769 or IgG-control mAb for 2 h. The wells were washed with PBS after incubation for 0.5 h, 1 h and 3 h, respectively. The cell number was counted after taking photograph (× 200 per optic field) with Image J software (National Institutes of Health, USA).

### Three-Dimensional (3D) spheroid homotypic adhesion assay

Multicellular spheroids were generated by the liquid overlay technique. 24-well tissue culture plates were coated with 250 μl of prewarmed 1% agarose (Roche, Switzerland) solution in serum-free medium. After the agarose was allowed to solidify and form a thin layer on the bottom of the dish, a single-cell suspension (2 × 10^4^/ml, 500 μl/well) of 143B cells (ab75769 and IgG group), Lenti-CD146 MG63 or pwpxl vector-only transfected MG63 cells were incubated at 37 °C in 5% CO_2_ for 0.5 h, 1 h and 3 h, respectively. Images were captured by bright-field microscopy and photographed in digital format. Three independent experiments were carried out.

### Heterotypic adhesion of OS cells to HUVECs

HUVECs (5 × 10^4^/ml, 100 μl/well) were placed on 96-well dishes for 24 h. Following this attachment, the wells were coated with a thin overlay of 2% BSA for 1 h, and 1 × 10^5^/well 143B cells (ab75769 and IgG group), Lenti-CD146 MG63 or pwpxl vector-only transfected MG63 cells were added to the plates with and without anti-CD146 monoclonal ab75769 or IgG control mAb (diluted 1:500) for 1 h. Wells were rinsed twice with PBS, and cells in each well were counted. Results are presented as the percentage of cells adhered from the total number of cells seeded. The experiment was repeated three times in triplicate.

### Endothelial cells were cultured in OS CM

143B cells (ab75769 and IgG group), Lenti-CD146 MG63 or pwpxl vector-only transfected MG63 cells (1 × 10^5^/ml, 8 ml/dish) were seeded into culture dishes, and the OS CM was collected at 80% fusions. OS CM or RPMI 1640 medium (as a control group) was used to culture HUVECs for 24 h to detect migration, permeability and tube formation, while the cells cultured at 72 h were used for proliferation assays.

### Endothelial permeability assays

12-well Transwell Boyden system (Sigma, CLS3414) was pre-coated with a layer of 0.05% gelatin (BD Biosciences), HUVECs were plated at 20,000 cells per well on membrane inserts (porosity 3 µm) and allowed to form monolayers. 500 μl RPMI 1640 medium supplemented with 10% fetal calf serum was added to the lower chamber. Then HRP-BSA (1 mg/ml, 10 μl, Sigma, USA) was added at the apical surface of the cells and was incubated for 12 h at 37 °C, in 5% CO_2_. The 100 μl CM of the upper and lower chambers were collected; following the 100 μl TMB chromogenic liquid (Sigma, USA) was added. Absorbance was read at 630 nm. The cell permeability was calculated: permeability rate = (OD value of below/OD value of above) × 100%. Experiments were performed at three times.

### Tube formation assay

24-well plates were coated with 200 μl Matrigel (BD Biosciences) following the manufacturer’s directions. After the appropriate treatments, HUVECs (1 × 10^6^/ml, 500 μl/well) re-suspended in complete medium were added to each well and incubated at 37 °C, in 5% CO_2_, for 12 h. Pictures were captured with bright-field microscopy. Only the number of closed tubes (means no defects in any tube walls) was counted and their diameters were measured after taking photograph (× 100 per optic field) with Image J software (National Institutes of Health, USA).

### Endothelial cells and OS, MG63 and 143B co-culture system

In order to test the expression of CD146 in the interaction between endothelial and OS cells, using the Transwell system of 3 μm pore size that could not pass the cell but could carry out metabolic exchange, we designed the following experiment: 1 × 10^5^/ml HUVECs in 150 μl complete medium were seeded to top well of a 12-well Transwell Boyden system (3 μm pore size, Sigma, CLS3414), and 1 × 10^5^/ml OS in 500 μl complete medium was added to the lower membrane inserts. After co-culture with 48 h, different groups of cells were collected for real-time qPCR and Western blotting, and CM from co-culture system and CM from primary culture condition were collected for ELISA. In addition, we also co-cultured MG63 and 143B for 24 h or 48 h, and the culture method and the number of seeded cells were the same, as mentioned above. The cells before co-culture were recorded as 0 h.

### ELISA

Dilute the standard substance according to the manufacturer’s instructions (R&D, USA) and add 100 μl per well into the ELISA plate for 1 h at 37 °C. After four washes using PBS supplemented with 0.05% Tween 20, Biotin-conjugated secondary antibody working fluid (R&D, USA) at 1:100 in PBS were added in each well for 30 min at 37 °C. Plates were washed four times, and then 100 μl of TMB substrate (Sigma, USA) were added in each well for 15 min at RT. The reaction was stopped by adding 50 μl of Stop Solution, and absorbances were read at 450 nm.

### Cell culture environment models

The OS and endothelial cells with a density of 1 × 10^4^/ml were placed on a 6-well plate for 48 h, and divided into 5 groups according to the culture environment, as follows: (1) Hyperoxia (HO). The oxygen concentration was adjusted to 30% and 5% CO_2_ through the three-gas incubator; (2) Low oxygen (LO). Oxygen concentration was 1%, 5% CO_2_; (3) High glucose (HG): DMEM containing 10% fetal bovine serum was used as the basic medium, and the final glucose concentration was 4500 mg/L; (4) Low glucose (LG). The final concentration of glucose is 1000 mg/L; (5) Normal. Cells were cultured in 5% CO_2_, 21% O_2_, 37 °C saturated humidity, and 2000 mg/L glucose concentration as a control group.

### Statistical analyses

SPSS 19.0 was employed to perform the statistical analysis (IBM, Armonk, NY, USA). All data are presented as the mean ± SEM of at least three samples. Repeated measure ANOVA, One-way ANOVA or Student’s t-test was applied to perform the statistical analysis. P < 0.05 was considered to indicate statistical significance.

## Result

### There is no CD146 expression in MG63

**Fig. 1:**
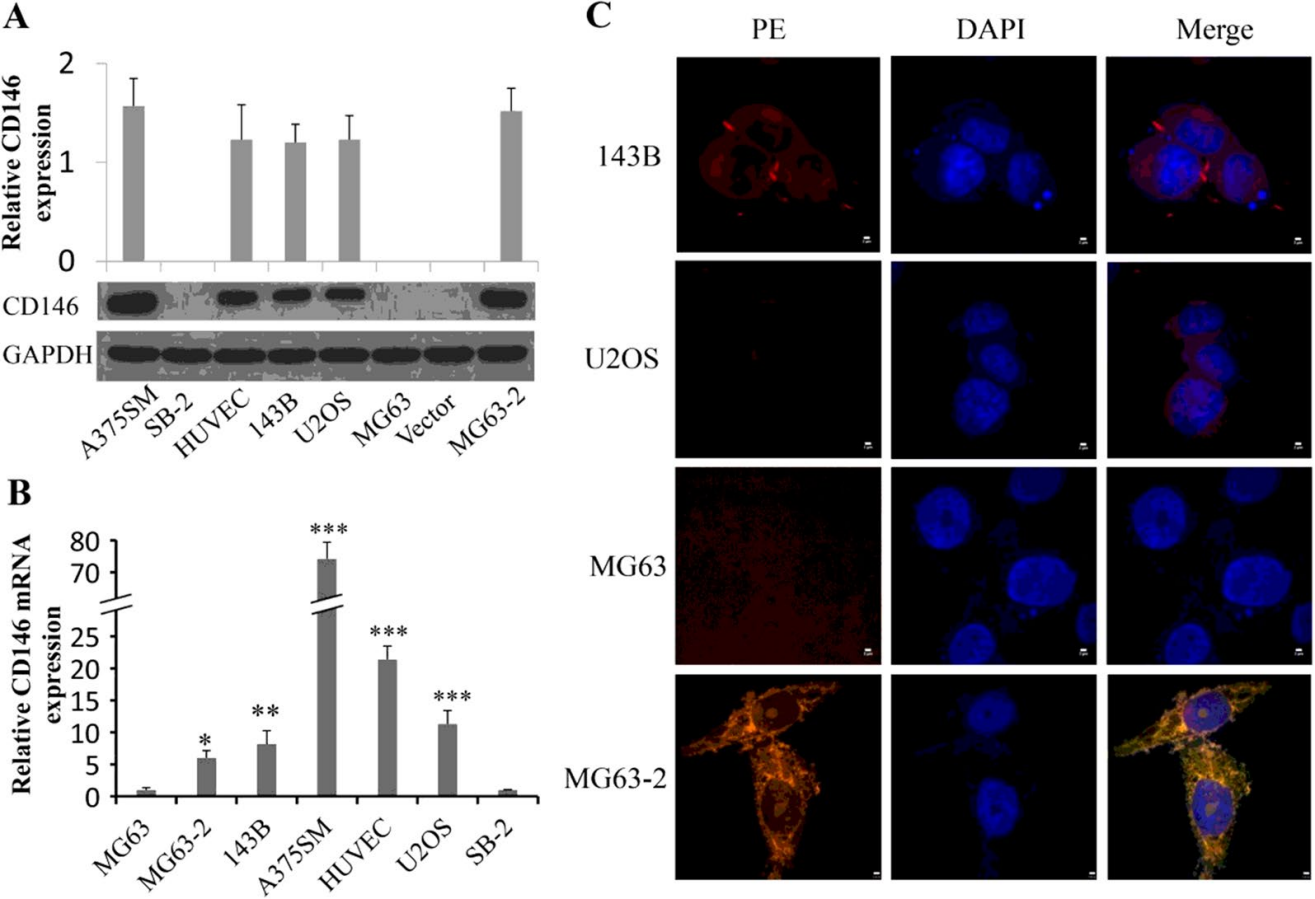
CD146 expression on human OS cells. The expression levels of CD146 were determined by Western blotting (**A**) or qPCR (**B**), and GAPDH served as an internal control. The A375SM and HUVEC were used as positive controls, and SB-2 as a negative control. For mRNA, CD146 was normalized to GAPDH first, and expression levels were compared to that of MG63 (set as 1). **C** Double-staining immunofluorescence and confocal microscopy analysis of CD146 expression and distribution in human osteosarcoma cells. The merged figures were the overlapping of red (CD146) and blue (nucleus) signals. The merged colour in MG63-2 was partially affected by the vector pwpxl-GFP (green, Additional file 1: Fig. S3C). Scale bar, 2 μm. *P < 0.05; **P < 0.01; ***P < 0.001

Whether MG63 cells positively express CD146, different literatures have different results. McGary et al. found MG63 did not express CD146 [7], while Schiano and co-workers showed the fully opposite result [22]. To verify the actually situation, we analyzed the expression of CD146 in MG63 by three different methods: Western Blotting, Immunofluorescence and sq-PCR. First, Western Blotting showed no CD146 positive strips appeared in MG63, either with anti-CD146 mAb ab134065 dilution of 1:200, 1:500 and 1:2000 (Additional file 1: Fig. S2A), or with anti-CD146 mAb ab75769 dilution of 1:1000 (Additional file 1: Fig. S2B, S2A; Fig. 1A). Then, we measured CD146 expression at gene level by sq PCR (Additional file 1: Fig. S2C), and no detectable band of CD146 mRNA could be explored in MG63. Similarly, no CD146 positive fluorescence (red color) was observed in MG63 by immunofluorescence assay (Fig. 1C). These results thus demonstrate that MG63 cells have no CD146 expression.

### CD146-expressed MG63 clones are selected for subsequent studies

After stable transfection of CD146 into MG63, two monoclonal colonies, named MG63-1 and MG63-2, were selected randomly to detect the effectiveness of transection by using methods of Western blotting and qPCR. The wild MG63 cells as a negative control group, including protein and gene levels. It should be noted that although MG63 does not express CD146, trace amounts of gene transcription can still be captured by qPCR. The results showed that MG63-2 expressed much more CD146 than MG63-1 and MG63 at both transcriptional and translational levels (Additional file 1: Fig. S3A, B). So we chose MG63-2 for further researches and verified the transfection effect by using Immunofluorescence. The results showed that the cells transfected with empty vector emitted green light (Additional file 1: Fig. S3C), while the CD146 transfected cells exhibited yellow color (Fig. 1C).

### CD146 expression levels are different in human OS cell lines

Expression of CD146 on U2OS, 143B and MG63-2 OS cells was determined at mRNA and protein levels (Fig. 1). It has been showed that CD146 expression was high in all the three types of OS cells at protein level and was moderate at mRNA level compared to the positive control HUVEC and A375SM cells (Fig. 1A, B). The results might indicate that the translation process of CD146 in these three cell lines was relatively more active. Interestingly, in immunofluorescence assay, we found that the CD146 distributed in the cytoplasm and membrane of the OS cells, which was different with other tumor cells like melanoma and prostate cancer cells, in which CD146 presence on the cell membrane [6] (Fig. 1C).

### Roles of CD146 in the progression of OS cells

**Fig. 2:**
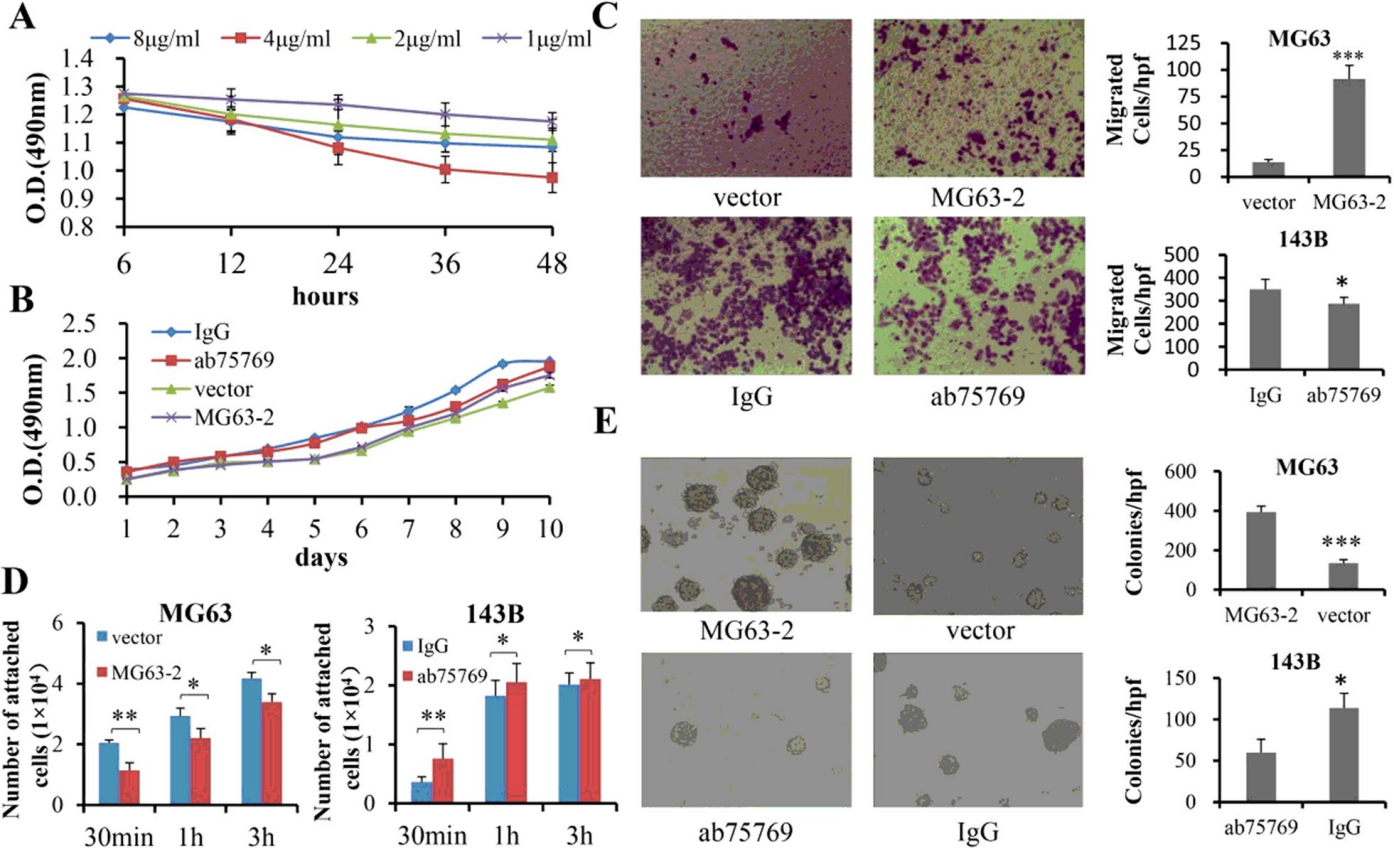
Effects of CD146 on progression of OS cells in vitro. **A** Inhibitory role of ab75769 in proliferation of 143B cells at four-tier concentrations for 48 h (n = 6). **B** The proliferation of MG63-2 and 143B treated was analyzed by MTT assay (n = 6). Vector-transfected MG63 and 143B treated with IgG served as blank controls. **C** The migration of MG63-2 and143B was detected by using Transwell system (n ≥ 4). **D** The adhesion capability between OS and type I collagen was quantitatively measured for 30 min, 1 h or 3 h, respectively (n = 5). **E** The matrix colony formation was observed at MG63-2 and 143B (n = 3). Unless otherwise special explanation, in this study, the vector referred specifically to MG63 empty vector group; and IgG, ab75769 acted on 143B cells. Scale: × 100. *P < 0.05; **P < 0.01; ***P < 0.001

In the CD146 positive expression of OS cells, we selected 143B as one of the subjects due to its high malignancy, chemoresistance and genomic instability [23]. In order to investigate the role of CD146 in the progression of 143B, we first explored the optimal inhibitory concentration of anti-CD146 mAb ab75769. The concentration of ab75769 was divided into 4 groups: 1, 2, 4 and 8 μg/ml. The results showed that 4 μg/ml group had the strongest inhibitory effect on the proliferation of 143B cells (Fig. 2A; F = 13.602, P < 0.001; 4 μg/ml: P = 0.070 vs. 8 μg/ml, P = 0.002 vs. 2 μg/ml, P < 0.001 vs. 1 μg/ml), so we chose this group for subsequent experiments and defined as “ab75769 group”.

After CD146 was inhibited by ab75769, the proliferative activity of 143B decreased (MTT: F = 140.192, P < 0.001, Fig. 2B; cell count: F = 8.028, P = 0.047, Additional file 1: Fig. S4A), as well as human mesenchymal stem cells [24]. However, ectopic overexpression of CD146 by lentivirus transfection (MG63-2) did not significantly increase the number of MG63 cells (MTT: F = 4.192, P = 0.601, Fig. 2B; cell count: F = 2.764, P = 0.172, Additional file 1: Fig. S4A), nor did it in melanoma [6].

Next the effect of CD146 on OS migration and invasion was analyzed. 143B cells treated with ab75769 exhibited significantly less motility and invasion than did IgG-treated cells (Additional file 1: Fig. S4B). Moreover, MG63-2 had a significantly 6.8-fold higher migration and 6.5-fold higher invasiveness than the vector-control group (Fig. 2C, Additional file 1: Fig. S4B). These results showed that CD146 can significantly enhance the malignant degree of OS.

In order to observe the effect of CD146 on adhesion of OS cells to ECM, we used OS cells to interact with type I collagen, the main component of ECM [25]. Figure S3C showed that the vector-control MG63 and 143B treated with ab76769 adhered very well to collagen-coated wells and exhibited spindle morphology with the extension of incubation time. In contrast, MG63-2 and IgG-control 143B cells were round and displayed poor ability to attach to collagen. Moreover, the quantitative data further confirm the above results (Fig. 2D). These data suggested that CD146 inhibited the adhesion of OS cells to ECM to facilitate OS cells to escape from the original location and metastasize to the distant.

Next, we used 3D spheroid assay, which can be used to mimic the growth of tumor cells in vivo [26], to detect the role of CD146 in the homotypic adhesion of OS cells. Additional file 1: Figure S3D showed that both MG63-2 and 143B formed spheroids, however, those process were disrupted when the cells were vector-transfected or treated by ab75769, respectively. The above conclusions revealed that CD146 could promote the formation of OS multicellular aggregates and tumor growth.

Since colony formation in matrigel reflects population dependence, proliferative and aggressive capacity of tumor cells, it can be used to evaluate the tumorigenicity in vitro [26], an indicator of tumor size and a prerequisite for intravascular clustering to distant metastasis. As shown in Fig. 2E, we observed that the number and size of MG63-2 clones were much higher than those of vector-control group. Similarly, 143B was observed to have many large clones, but they were weakened by the anti-CD146 mAb ab75769. Therefore, we concluded that CD146 enhanced the tumorigenicity of OS cells in vitro.

In summary, it is presumed CD146 promotes tumor escape from the original site and early metastasis to surrounding tissues in vitro by increasing the migration and invasion of OS cells, and reducing the heterotypic adhesion between tumor cells and ECM. Furthermore, it is believed that the number of surviving cells attacked by immune cells in blood vessels can be increased by enhancing the homotypic adhesion and tumorigenicity of tumor cells, which is conducive to the distant metastasis.

### Effects of OS malignancy on CD146 expression

**Fig. 3:**
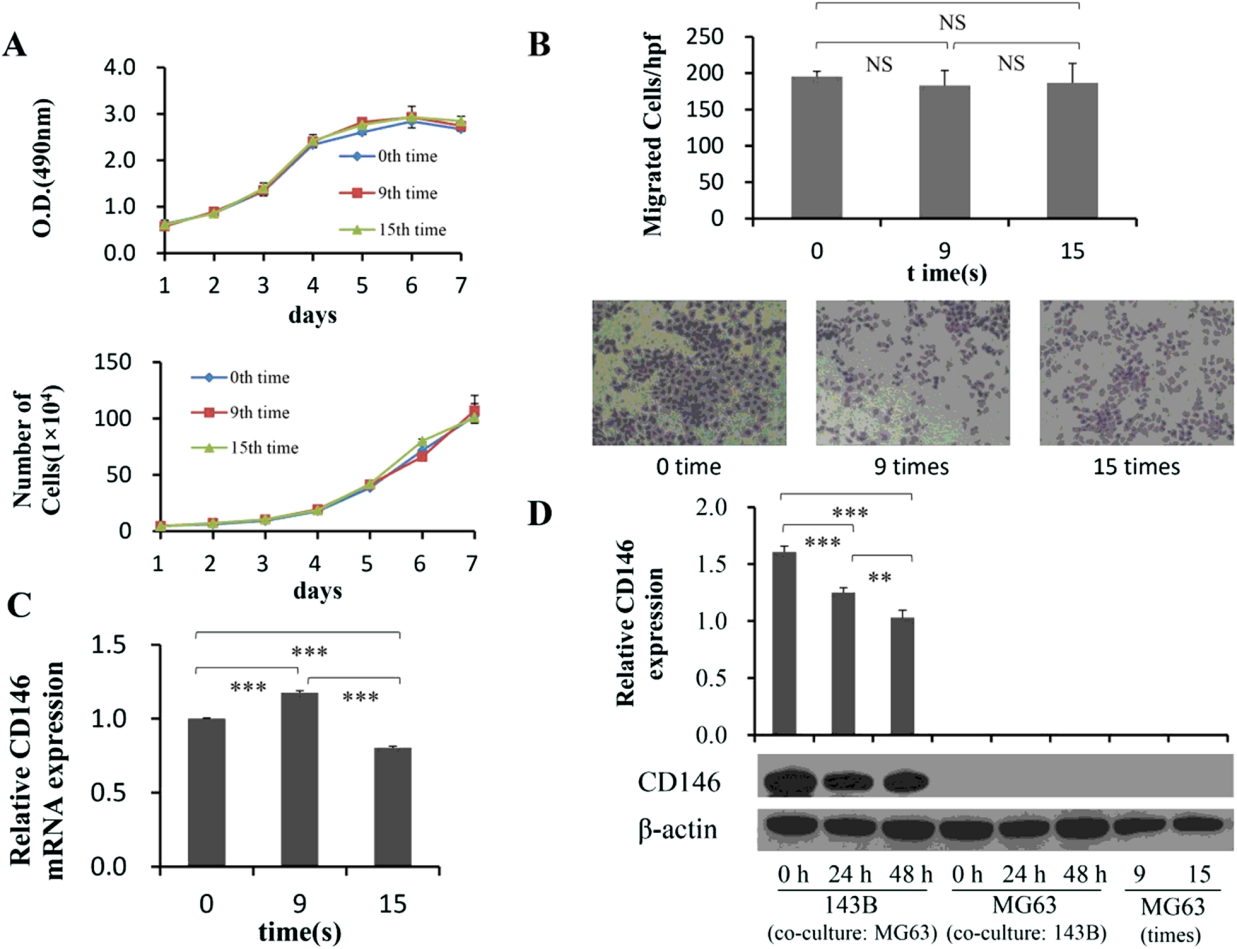
Effects of OS malignancy on CD146 expression, including cycle migration and co-culture assay in vitro. **A** The proliferation of MG63 cells that come from the 0th, 9th or 15th time repeated migration was observed by MTT and Cell counting assays (n = 6). **B** The migration ability of MG63 cells, which were collected at the 0th, 9th, and 15th time migration, respectively, was measured by using Transwell system (n = 5). The migrated cells were counted by Image J software. The expression of CD146 mRNA (**C**) and protein (**D**) in MG63 cells derived from the 0th, 9th, and 15th time migration were detected by qPCR and Western blotting (n = 3). **D** In addition, 143B cells were co-cultured with MG63 cells for 0, 24 and 48 h to detect the CD146 expression, respectively (n = 3). Scale: × 200. **P < 0.01; ***P < 0.001

To investigate the correlation between the malignant transformation of OS and the expression of CD146, first, a cyclic migration experiment (see “Materials and Methods”) was designed to observe whether the malignant of MG63 cells and the corresponding CD146 expression would change. The results showed that after nine or fifteen time migrations, the proliferation of MG63 cells had hardly changed comparing to the initial time (Fig. 3A), both by MTT (F = 3.635, P = 0.052) and cell counting assays (F = 1.550, P = 0.287). Similarly, the number of MG63 cells migrated through the Transwell membrane was also no dramatic differences among cells from the 0th, 9th and 15th time migration (Fig. 3B).

Then we further examine the CD146 expression by using qPCR and Western blotting (Fig. 3C, D). Compared with non-migrated cells, the process of repeated migration in the early stage stimulated the mRNA expression of CD146 in MG63 cells, peaked at 9th time (Fig. 3C), and then declined and even suppressed at 15th time (Fig. 3C). Unlike mRNA, there was no CD146 protein expression in MG63, no matter how many times the migration was repeated (Fig. 3D). The above results demonstrated that cyclic migrations of MG63 cells had not altered their biological characteristics, and not affected the CD146 expression either. It is also pointed out that the compositional change of tumor heterogeneity is a passive process, and the malignancy tumors do not be easily changed in the external environment, but probably evolve under the effect of immunity or drugs in the body.

Next, to address whether the expression of CD146 is regulated by the interaction of OS cells with different malignant grades, we co-cultured MG63 with 143B cells, and found that the protein expression of CD146 in MG63 did not turn positive (Fig. 3D), indicating that 143B could not activate the post-transcriptional expression of CD146 in MG63 cells. Interestingly, the levels of CD146 protein and mRNA expression in 143B cells were significantly decreased at 24 h and 48 h after co-culture (Fig. 3D, Additional file 1: Fig. S5), suggesting that MG63 might produce some factors that inhibited CD146 expression of 143B by participating in inhibition of both transcriptional and translational process.

### CD146 in OS cells mediates endothelial behaviors

**Fig. 4:**
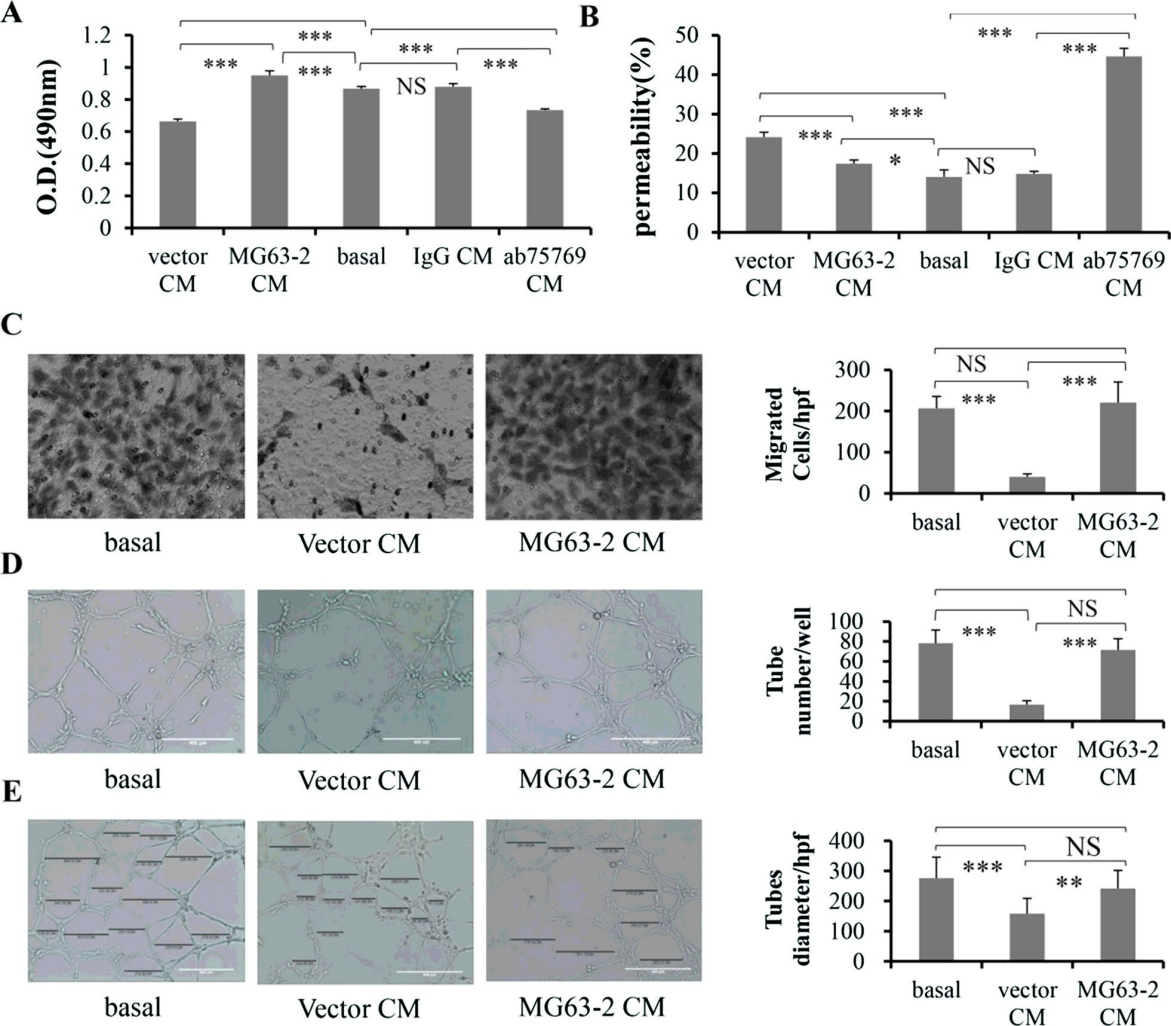
Effects of OS CM on progression of HUVECs in vitro. The proliferation (**A**) and permeability (**B**) of HUVECs were analyzed by MTT and Endothelial permeability assays, after HUVECs were treated with OS CM for 72 h or 24 h, respectively. (**C**) The migration capability of HUVECs was measured using a Transwell system after HUVECs were cultured in MG63 CM for 24 h. Scale: × 100. **D**, **E** Tube formation and diameter size were analyzed after HUVECs were incubated in MG63 CM for 24 h. The quantification of the tube length and diameter was done by Image J software and is presented in the histogram right. Bar = 400 μm. *P < 0.05; **P < 0.01; ***P < 0.001. The data were average results of experiments repeated for six (**A**), three (**B**, **D**), four (**C**), and at least eight (**E**) times, respectively

OS CM was used to treat HUVECs to analyze the effect of CD146 in OS cells on the biological characteristics of endothelium. The HUVECs CM served as a control group, recorded as “basal”. After 72 h of culture, CM from the vector-control MG63 and 143B CM treated with ab75769 significantly inhibited HUVECs proliferation, compared with basal. Unexpectedly, MG63-2 CM increased proliferation of HUVEC, but not 143B with IgG (Fig. 4A).

The effect of OS CM was tested on HUVEC permeability. The ab75769 promoted the permeability of HUVECs with an effect similar to that observed with vector CM. However, the MG63-2 or 143B IgG CM just slightly affected the permeability, especially for latter (Fig. 4B). Similarly, both of them did not alter the HUVECs migration and the number and diameter of tubes formed compared with the basal, either. On the other hand, the vector CM and ab75769 CM decreased the above biological parameters of the endothelium effectively (Fig. 4C–E, Additional file 1: Fig. S6A–C).

**Fig. 5:**
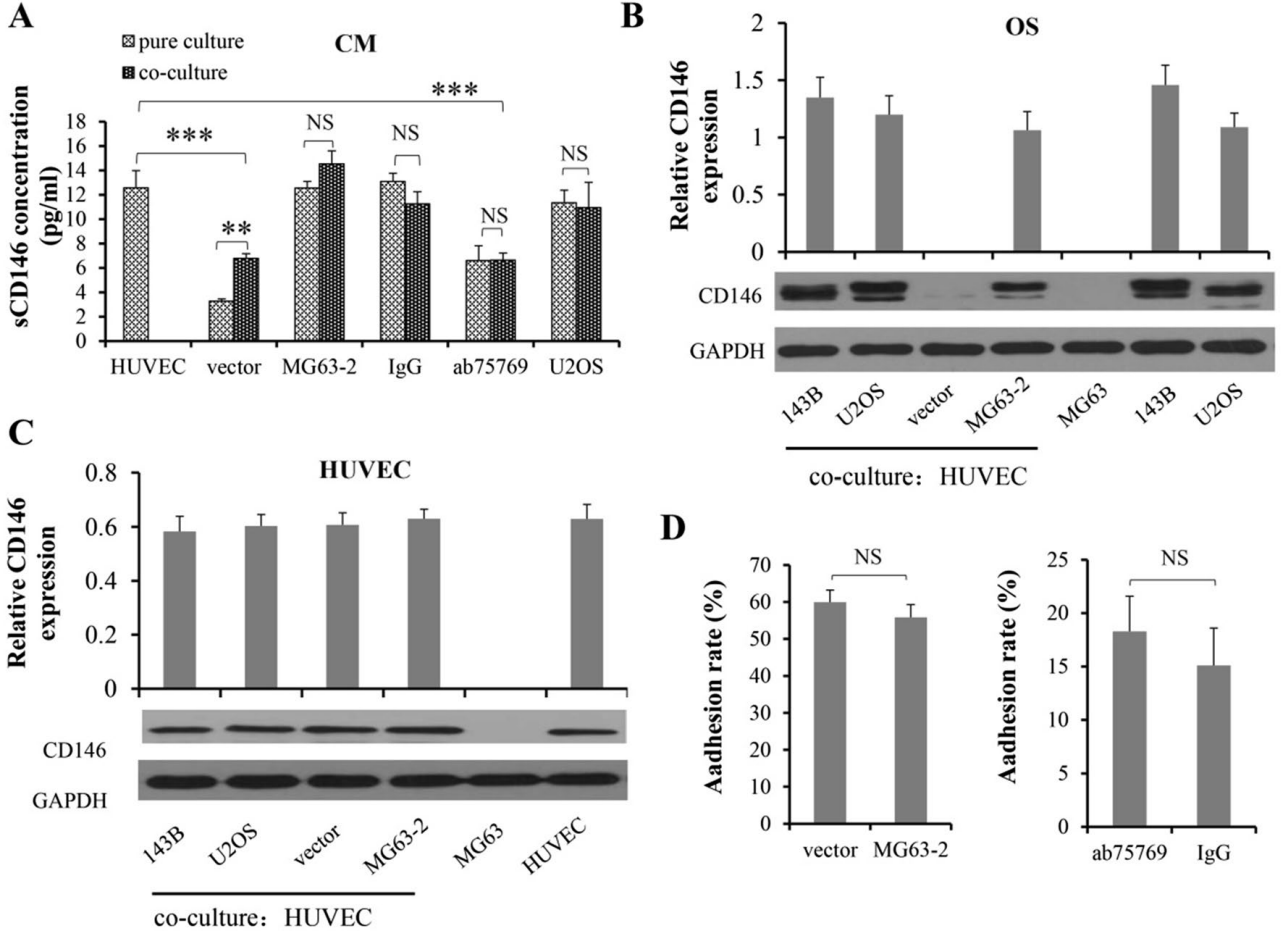
Relationship between CD146 and the interaction of OS and HUVECs. **A** The sCD146 levels in the CM from single or co-culture system were measured. The protein expression of CD146 in OS (**B**) or HUVECs (**C**) were observed by Western blotting before and after OS cells were co-cultured with HUVECs. **D** The role of CD146 in heterotypic adhesion rates between them was detected. GAPDH was used as internal control. **P < 0.01, ***P < 0.001, n = 3

Next, we further detected the sCD146 concentration in basal and co-culture CM of OS and HUVECs, and found that sCD146 in vector and ab75769 co-culture CM was significantly decreased compared to basal, while the other two groups did not (Fig. 5A). These results suggested although the effect of CM from MG63-2 and 143B treated with IgG mAb on endothelial behaviors was not exactly the same, targeting CD146 by reducing the concentration of sCD146 in the CM effectively inhibited endothelial proliferation, migration, the tightness of endothelial connections, the number and diameter of tube-like structure, which is consistent with the report of Stalin et al. [27].

### The mutual contact between OS and endothelium is almost unrelated to CD146 expression

First, we detected the concentration of sCD146 in the CM before (i.e. pure culture of OS) and after co-culture of OS with HUVECs (Fig. 5A). Compared with OS pure medium, co-culture with endothelium did not increase the concentration of sCD146, except for the vector group, which might be because the sCD146 produced by HUVECs during co-culture increased the total concentration in the CM.

Next, we used the Transwell system to co-culture the OS and HUVECs for 48 h, and found that the interaction between OS cells and HUVECs did not significantly alter the expression of CD146 in OS cells, both protein and gene levels (Fig. 5B; Additional file 1: Fig. S7); nor did it change the content of CD146 protein in HUVECs (Fig. 5C). Then, in turn, we addressed the effects of CD146 on heterotypic adhesion between OS cells and HUVECs. After co-incubation for 1 h, the results showed that CD146 did not affect the adherence rate, regardless of CD146 transfection group or anti-CD146 mAb group (Fig. 5D).

Overall, these results indicated that OS co-cultured with endothelium hardly affected the CD146 expression. Interestingly, CD146 also did not work on the heterotypic adhesion between them.

### The expression of CD146 in different cells has different response to culture environment

**Fig. 6:**
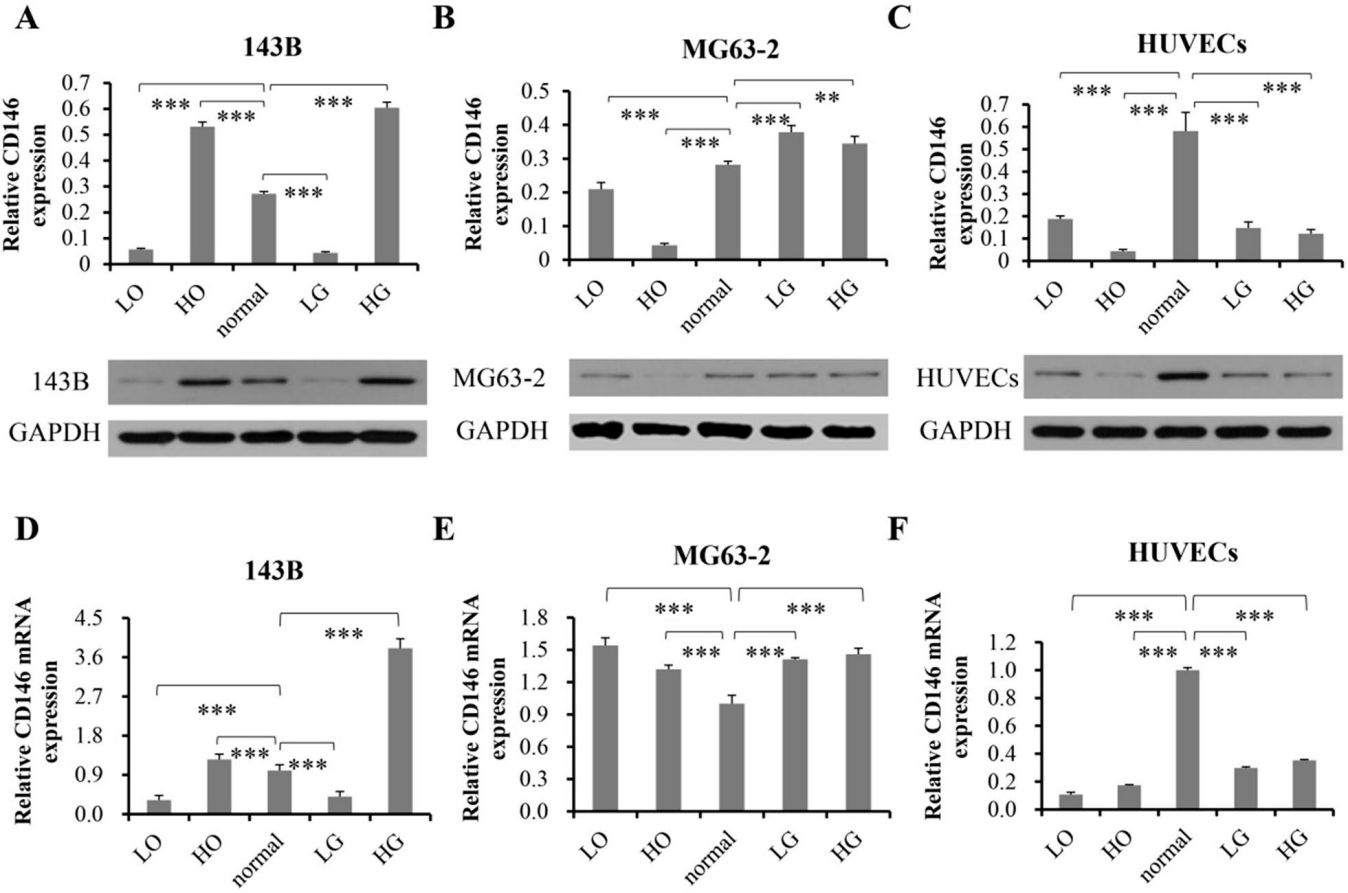
The effect of different culture environment on the expression of CD146 in OS cells and HUVECs. The protein expression of CD146 in 143B (**A**), MG63-2 (**B**) or HUVECs (**C**) were observed by Western blotting. The mRNA expression of CD146 in 143B (**D**), MG63-2 (**E**) or HUVECs (**F**) were observed by qPCR. They cultured in different culture environments, including hyperoxia (HO), low oxygen (LO), high glucose (HG) and low glucose (LG). Normal medium was used as a control. **P < 0.01, ***P < 0.001, n = 3

Since CD146 plays an important role in OS progression and regulation of endothelial behaviors, we wonder the response of CD146 in OS and HUVECs to different culture environments, including hyperoxia (HO), low oxygen (LO), high glucose (HG) and low glucose (LG). The data indicated that the expression of CD146 in different cells was diverse under different culture conditions, even in MG63-2 and 143B that are both OS (Fig. 6). Specifically, LO reduced the expression of CD146 protein in MG63-2 and 143B OS, while HG increased the content of CD146; The CD146 expression in 143B was increased under HO and decreased under LG, while the result was the opposite for MG63-2 (Fig. 6A, B, D, E); Compared with normal condition, the expression of CD146 in HUVECs was decreased in other culture environments, including LO, HO, LG and HG (Fig. 6C, F). In HO and LO environment, the CD146 protein expression of MG63-2 was lower than that of the normal group, while the mRNA level was higher, presumably due to the low efficiency of protein translation (Fig. 6B, E).

## Discussion

There was a different voice about whether MG63 expressed CD146. McGary and co-workers found no expression of CD146 protein in MG63 by using ABX-MA1 anti-CD146 mAb and Western blotting [7]. However, Schiano and colleagues proved that MG63 could express the CD146 by using three kinds of experimental ways: immunofluorescence, PCR and fluorescence-activated cell sorting (FACS) [22]. To clear this controversy, we designed the three ways, sq-PCR, Western and immunofluorescence, and used two kinds of anti-CD146 mAb, ab134065 and ab75769, to determinate if MG63 cells expressed CD146. It has been shown that CD146 expression is negative in MG63 cells, which is consistent with the conclusion of McGary et al. [7]. In addition, because Schiano et al. did not provide information on the anti-CD146 antibody and CD146 primers used in the paper [22], including its Reference Sequence NCBI or the relevant references cited, we hardly repeat their experimental results.

Since MG63 is low malignant and does not express CD146, we attempted to express CD146 ectopically in MG63 by lentivirus transfection, and applied CD146 mAb to 143B OS cell line with high malignancy to jointly observe the effect of CD146 on OS progression. We found that CD146 improved the ability of OS remote metastasis by enhancing the migration, invasion and the probability of OS cells escaping from in situ via reducing the heterotypic adhesion between OS and ECM. Furthermore, CD146 enhanced the tumorigenicity of OS by increasing the homotypic adhesion and clonal ability of single cells, although it had little effect on the proliferation of OS cells. Therefore, we systematically demonstrate for the first time that CD146 directly promote the progression of OS by strengthening the metastasis and tumorigenicity in vitro. Similar results have also been reported in other tumors, such as melanoma [6, 28], prostate cancer [6, 29] and breast cancer [30, 31], which suggest that CD146 may be a common potential target for tumor therapy.

However, up to now, few studies have focused on whether tumor progression affects the expression of CD146. So we have innovatively designed cyclic migration assay based on tumor heterogeneity and co-culture of OS cells with two different malignancies. Surprisingly, the results showed that the multiple migrations of MG63 cells could not enhance their proliferation and migration, nor could it turn the expression of CD146 positive, even after co-culture with 143B. So it is obvious that repeated migration of tumor cells cannot reduce the heterogeneity and malignancy of themselves in vitro, which also proves that the adaptive cloning of tumor cells is mainly caused by the screening of drugs or immune factors in vivo [32]. Compared to primary OS, for instance, a proinflammatory FABP4 + macrophages and lower osteoclasts infiltration are noticed in recurrent and lung metastatic OS lesions [33].

Tumor growth needs angiogenesis and nourishment. CD146, as a novel endothelial biomarker [34], plays an important role in maintaining vascular function [4, 5]. We observed that sCD146 in OS CM regulated endothelial cell proliferation, permeability, migration, vascular number and diameter. The decrease of sCD146 in OS CM increased the endothelial permeability, which is consistent with the conclusion that CD146 knockout led to the destruction of blood–brain barrier reported by Chen et al. [35]. In addition, down regulating the expression of CD146 in OS CM inhibited the proliferation, migration and lumen formation of endothelial cells, which is similar in pancreatic cancer and melanoma [27]. Considering that the endothelium in OS is in a state of high proliferation [36], so it can be concluded that targeted inhibition of CD146 can effectively assist in the treatment of OS by reducing angiogenesis and increasing the penetration of chemotherapy drugs, although it does not affect the heterotypic adhesion between OS and endothelium, which may be due to the fact that co-culture of them did not change their expression of CD146.

Tumors are generally in a state of hypoxic [17, 18]. Interestingly, hypoxia caused a decrease in the expression of CD146 in the OS and endothelium, just like in stem cells [37], which seems to contradict the conclusion that CD146 promotes the progression of OS. We speculate that CD146 promotes metastasis, tumorigenesis and angiogenesis in the initial stage of OS. After it grows to a certain volume, the decreased CD146 due to internal hypoxia in turn actively coordinates the adaptive slowdown of tumor growth and avoid thrombosis [38], and the saved nutrients supply the growth of peripheral tumor. Moreover, the increase of CD146 in OS under HG condition may be one of the potential factors for the increased risk of cancer in diabetic patients. Given the global epidemic of diabetes, targeted inhibition of CD146 may prevent both cancer occurrence in diabetics and the onset of diabetes in cancer patients, which will translate into a substantial socioeconomic benefit.

## Conclusions

In this study, we systematically proves that the CD146 is directly associated with the malignant progression of human OS by positively regulating of OS cell migration, invasion, single cell cloning and homotypic adhesion in vitro. Inhibition of CD146 enhances the heterotypic adhesion of OS cells and the permeability of endothelial cells, and reduces the proliferation, migration, lumen formation and diameter of endothelial cells. However, co-culture of OS and HUVECs do not change the expression of CD146, and MG63 cells cannot change their own heterogeneity and CD146 expression after multiple migrations. Since HG can increase the expression of CD146 in OS, interestingly, we can speculate that this may also be one of the reasons for the mutual risk factors between diabetes and some cancers, such as pancreatic cancer, liver cancer and melanoma, and so on. The above hypotheses, of course, still need to be verified by studies in vivo. These results suggest that CD146 is a potential diagnostic marker and therapeutic target for OS, especially in patients with diabetes.

## Supplementary Information

Supplementary file1 (DOCX 1179 KB)

## Data Availability

The data presented in this study are available on request from the corresponding author.

## Abbreviations

MCAM/CD146: Melanoma cell adhesion molecule
OS: Osteosacoma
mAb: Monoclonal antibody
GFP: Green fluorescent protein
GAPDH: Glyceraldehyde-3-phosphate dehydrogenase
PCR: Polymerase chain reaction
sq-PCR: Semi-quantitative PCR
qPCR: Quantitative PCR
HUVECs: Human umbilical vein endothelial cells
HO: Hyperoxia
LO: Low oxygen
HG: High glucose
LG: Low glucose
CM: Culture medium
ECM: Extracellular matrix
