# Supplementary material for: Recognize the role of CD146/MCAM in the osteosarcoma progression: an in vitro study
Source: Cancer Cell Int. 2021 Jun 8;21:300. doi: 10.1186/s12935-021-02006-7 (PMC8186124; doi:10.1186/s12935-021-02006-7)
Supplement: Supplementary file 1 — Supplementary file1 (DOCX 1179 KB) [file 12935_2021_2006_MOESM1_ESM.docx]

**Additional file 1**

**Recognize the role of CD146/MCAM in the osteosarcoma progression**

Xing Lei^1^, Kewei Wang^2^, Wenbo Wang^3^, Hao Jin^3^, Wenguang Gu^3^, Zhiguo Chen^1^, Wei Wang^1^, Kaituo Gao^1^, and Huan Wang^3*^

1. Department of Orthopedic Surgery, Linyi People’s Hospital, Linyi 276000, China

2. Center for Endemic Disease Control, Chinese Center for Disease Control and Prevention, Harbin Medical University, 157 Baojian Road, Harbin 150081, China

3. Department of Orthopedic Surgery, the First Affiliated Hospital, Harbin Medical University, 23 Youzheng Street, Nangang District, Harbin 150001, China

* Correspondence: [m4116@hrbmu.edu.cn](mailto:m4116@hrbmu.edu.cn) (H.W.)；Tel.: +86045185555801

LX6990064@126.com (X.L.); [kkww02052015@hotmail.com](mailto:kkww02052015@hotmail.com) (K.W.); wangwenbo@hrbmu.edu.cn (W.W.); 280798492@qq.com (H.J.); guwen-guang@hotmail.com (W.G.); flsh99@163.com (Z.C.); lybone@126.com (W.W.); lylbcdhy@163.com (K.G.); m4116@hrbmu.edu.cn (H.W.)


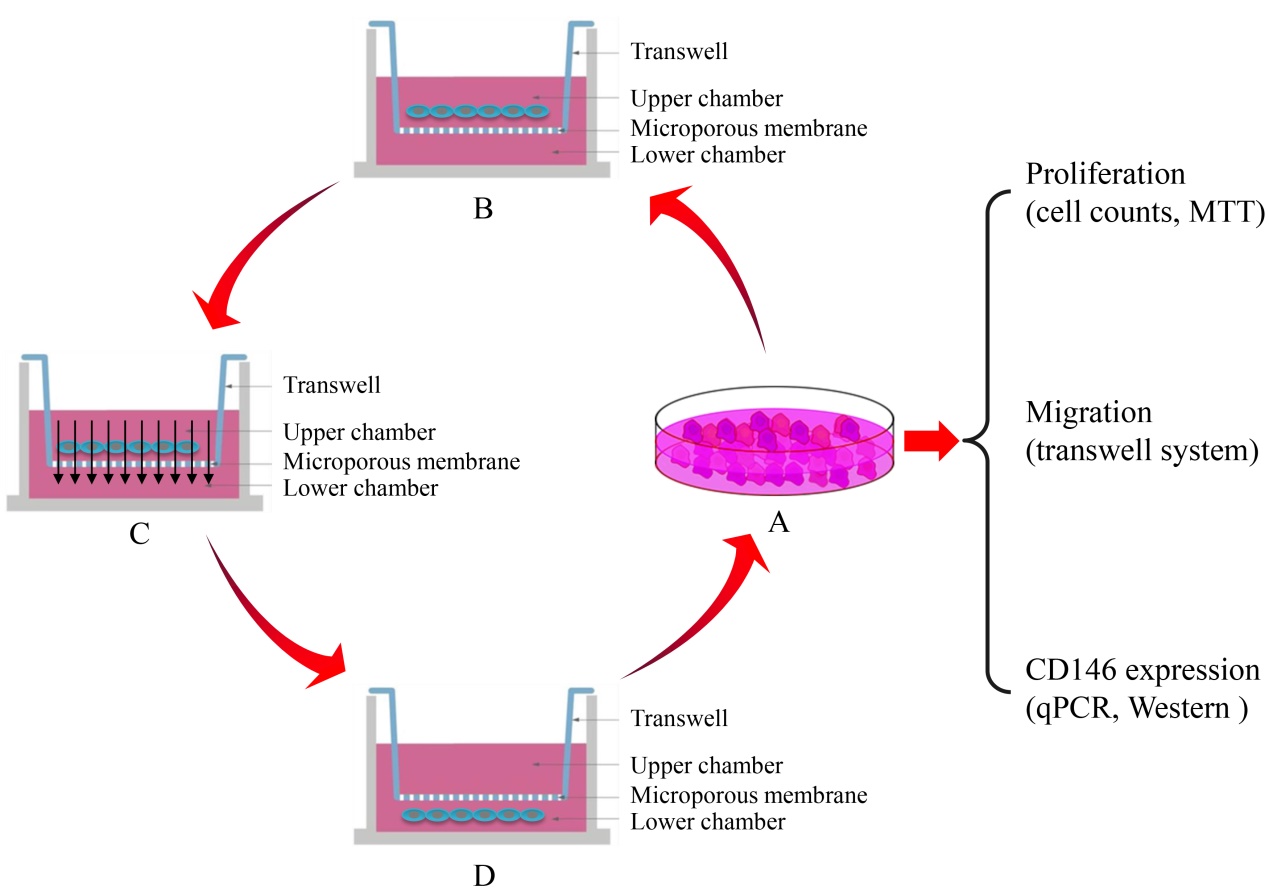


**Fig. S1.** Schematic diagram of cyclic migration assay. (A) Cell culture. (B) The cells were placed in the upper layer of Transwell chamber. (C) The upper cells are migrating to the lower layer. The cells in the lower layer were retained (D) and continue to the next round of culture (A) until 9 or 15 cycles were repeated, respectively.


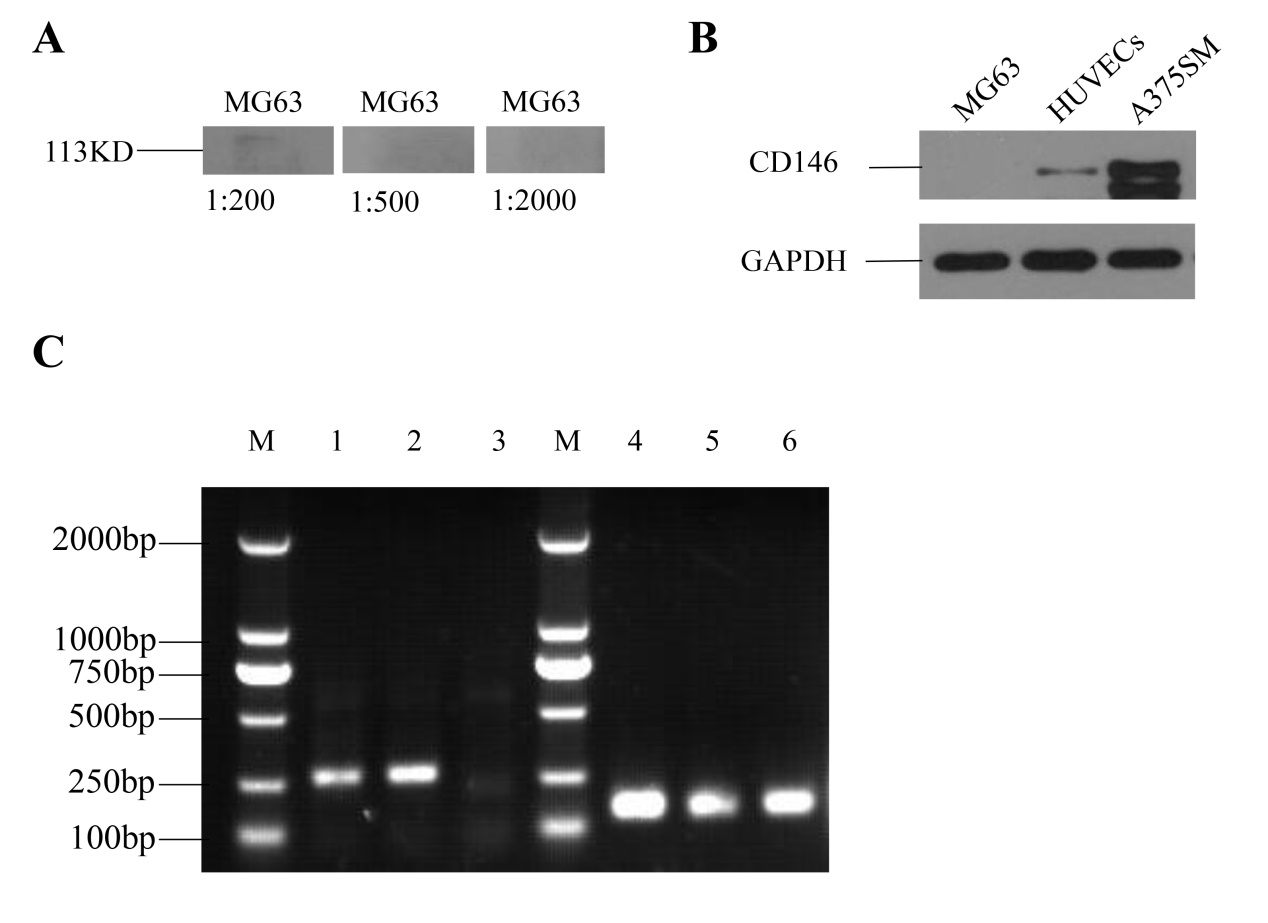


**Fig. S2.** CD146 expression in MG63 cell line. (A) The detection of CD146 expression in MG63 with CD146 mAb ab134065 at the concentration of 1:200, 1:500, 1:1000 respectively. (B) Verification the expression of CD146 by using another type of mAb ab75769 (1:500). (C) The expression level of CD146 was further analyzed with semi-quantitative PCR. Lane 1-3 referred to amplified product of CD146 mRNA. These numbers sequentially represents HUVECs, A375SM and MG63, respectively. Lane 4-6 showed GAPDH mRNA as internal controls. These numbers represent the same cells as 1-3. M: DNA Marker. A375SM and HUVECs were as positive controls.


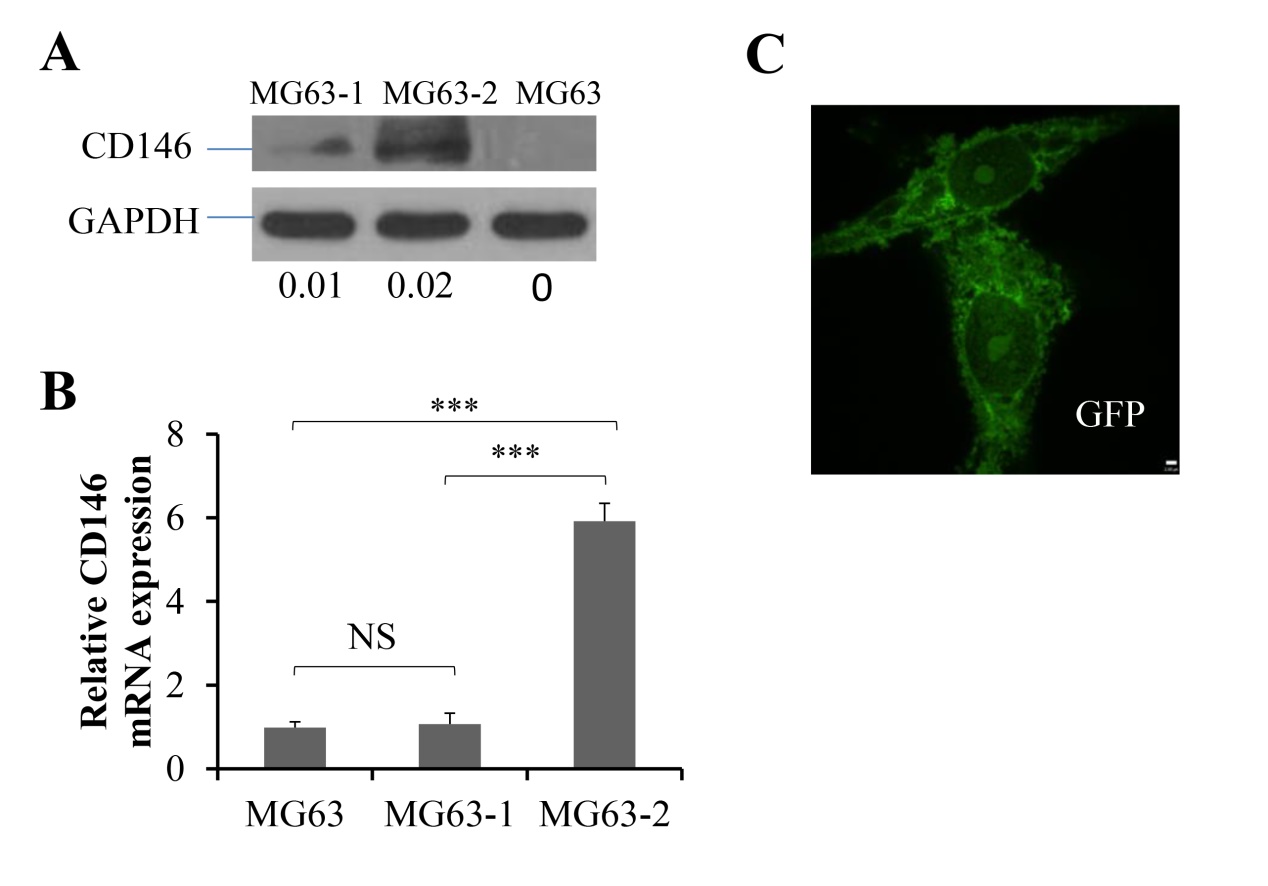


**Fig. S3.** The expression of CD146 in transfected MG63. (A) Determination of CD146 protein expression in MG63-1 and MG63-2 with CD146-mAb ab75769 (1:500) showed band at Mr 113kDa with relative gray value of 0.01 and 0.02, respectively. (B) Real-time qPCR was used to detect the relative expression level of MG63-1 and MG63-2 compared to MG63. (C) The green fluorescence emitted by pwpxl-GFP was observed in MG63 cells transfected with empty vector by using Immunofluorescence. The data were average results of experiments repeated for three times. Scale: 4μm, ***, P<0.001.


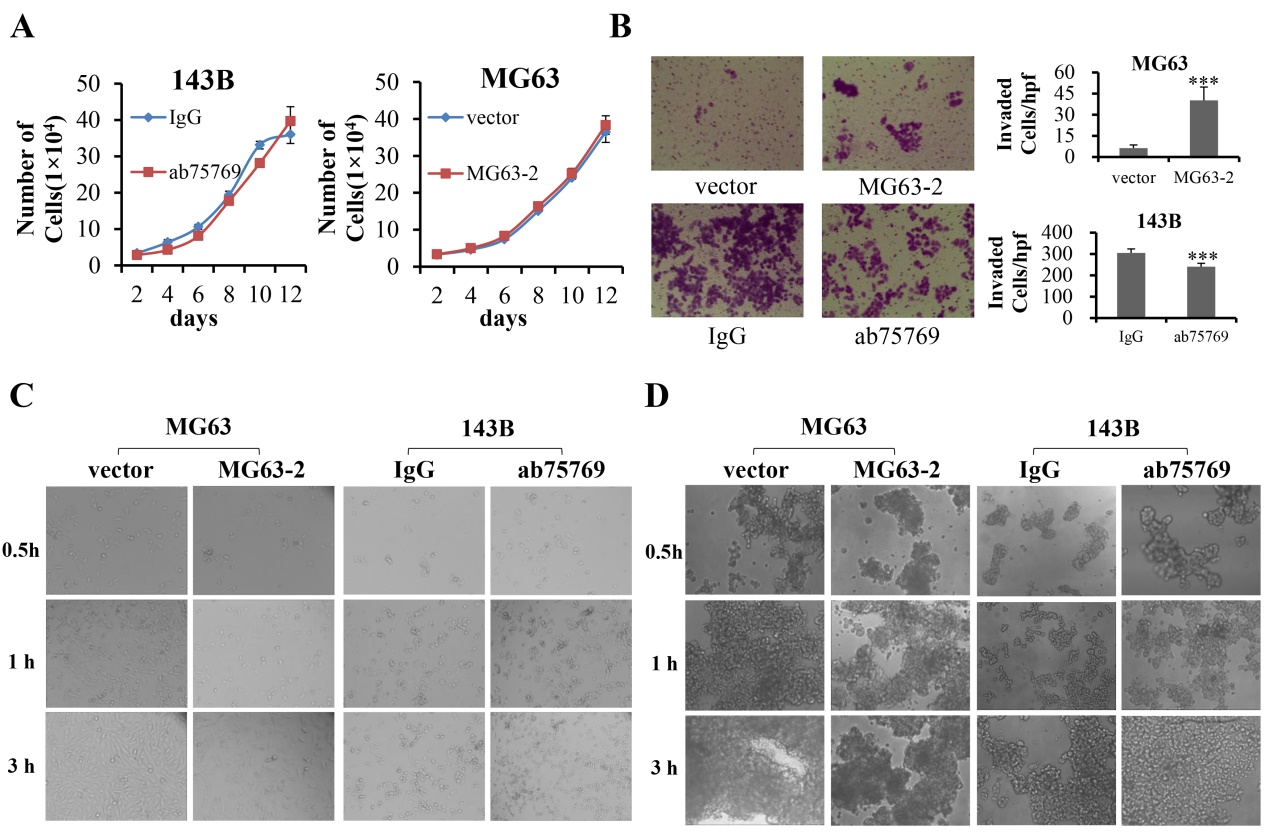


**Fig. S4.** Effects of CD146 on progression of OS cells *in vitro*. (A) The proliferation of MG63-2 and 143B was analyzed by Cell counting assay. Vector-transfected MG63 and 143B treated with IgG served as blank controls (n=3). (B) The invasion of MG63-2 and143B was detected by Transwell assay by measuring the number of cells across the Transwell member coated with Matrigel (n=5). The invasive cells were counted by Image J software and are represented in the graph right. (C) The adhesion capability between OS and typeⅠcollagen was measured after MG63 were transfected with CD146 or 143B cultured in ab75769 for 30min, 1 h or 3 h, respectively. (D) The spheroid formation was observed at MG63-2 and 143B, but blocked at MG63 transfected with vector or 143B treated with ab75769. Scale: ×100. ***, P <0.001.


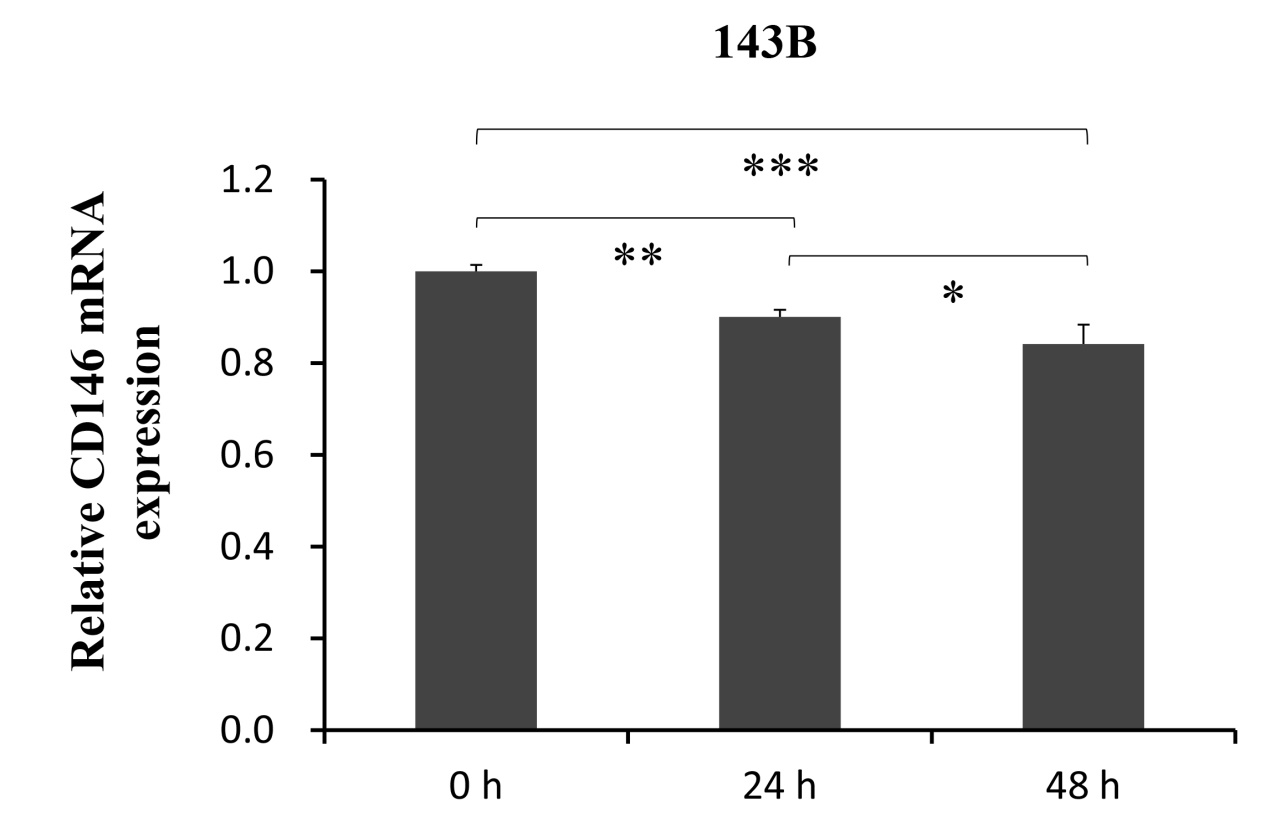


**Fig. S5.** The mRNA expression of CD146 in 143B cells, after they were co-cultured with MG63 cells for 0, 24, and 48 h, respectively. CD146 was normalized to GAPDH ﬁrst, and expression levels were compared to that of 0 h (set as 1).


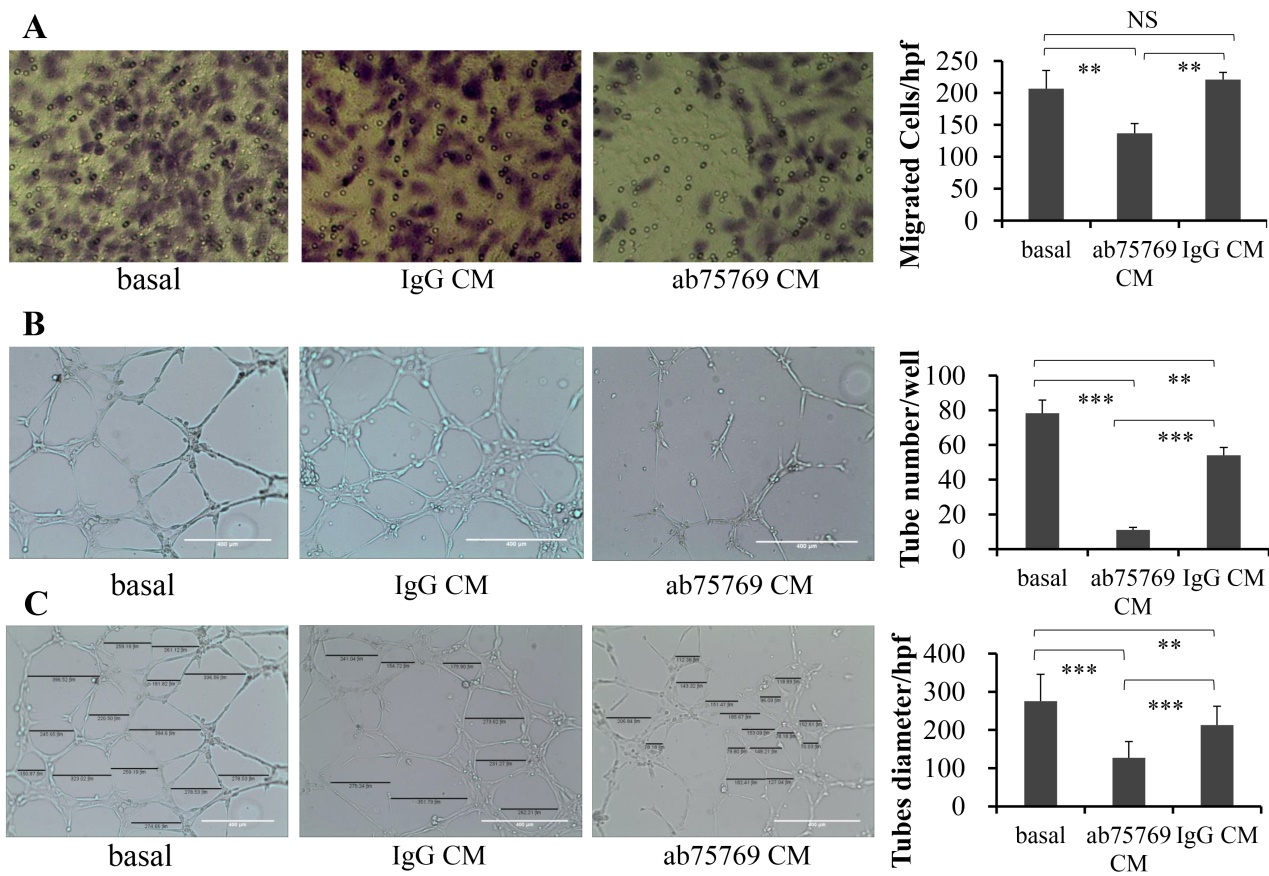


**Fig. S6.** Effects of OS CM on progression of HUVECs *in vitro*. The role of 143B CM in migration of HUVECs (A), capillary-like numbers (B) and diameter size (C) was evaluated. Vector CM and IgG CM were used as blank controls, respectively. Scale: ×100, bar=400μm. **, P <0.01; ***, P <0.001. The data were average results of experiments as means±SD repeated for four (A), three (B), and at least ten (C) times, respectively. The average tube numbers and diameters in each section of Matrigel plugs were quantiﬁed by Image J software.


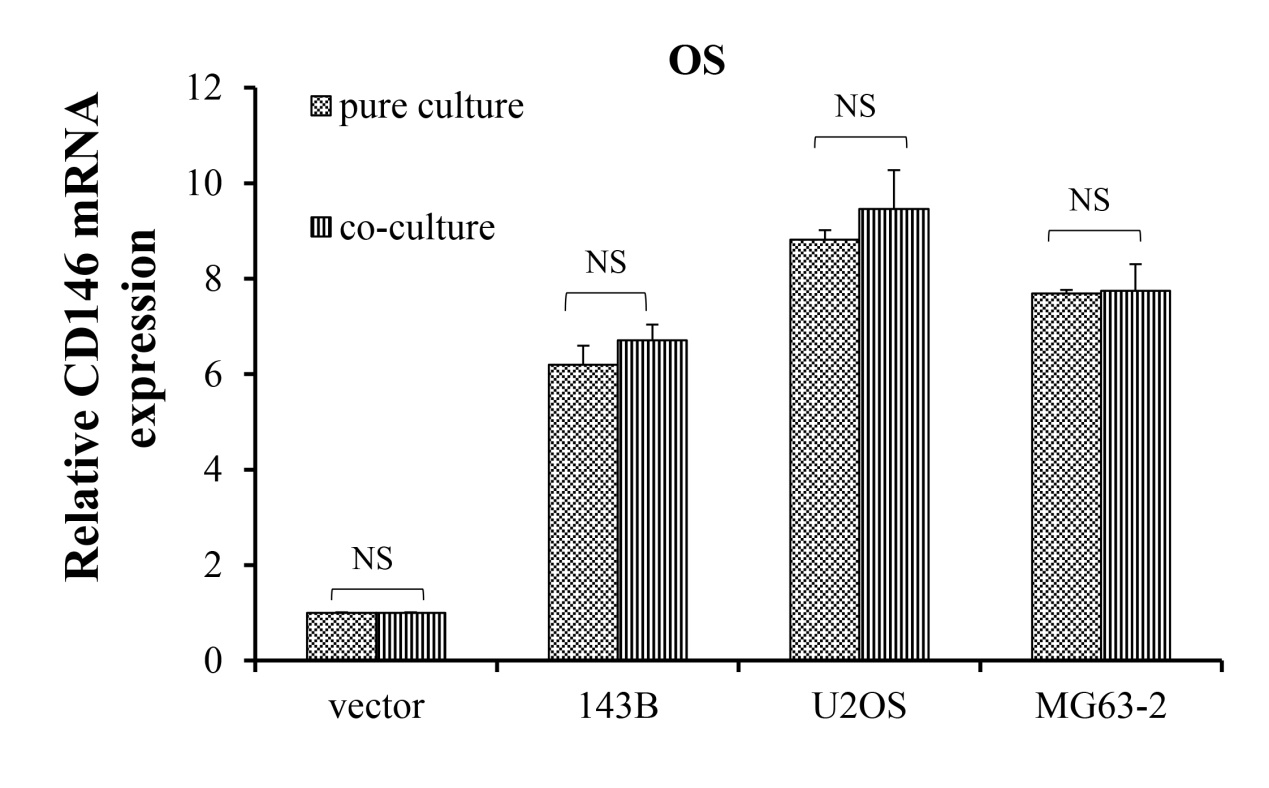


**Fig. S7.** qPCR analysis of the CD146 mRNA expression in OS from pure or co-culture system. CD146 was normalized to GAPDH ﬁrst, and expression levels were compared to that of vector pure cultured (set as 1).
